# Notes and Comments

**Published:** 1899-11

**Authors:** 


### Notes and Comments.[1]

Death from cutting a Wisdom-Tooth.—M. Heydenreich reported the case of a man, thirty-three years of age, brought to his clinic and said to be suffering from mumps. There was high and persistent fever, rising to 104° F., with agitation, delirium, stiffness of the jaws, and swelling over the right parotid extending into the neck. When M. Heydenreich saw the patient, on the third day of the grave symptoms, the condition seemed to have improved. The temperature was from 102.5° to 100.4°, consciousness had returned, and the swelling was strictly limited to the angle of the right jaw. The patient could open his mouth, and a drop of pus escaped by the jaw. All the teeth were there. It was certainly a case of suppurative osteitis of the inferior maxilla, due to the eruption of a wisdom-tooth. There was not at this time any indication calling for operative measures. The next day, however, the patient became semi-prostrate, and in the evening the temperature rose to 104.9° F.; on the fifth day he was taken in a moribund condition to the hospital. There was complete left hemiplegia. A free incision was made by means of the thermal cautery as far as the zygoma, but no pus was found. He died next day at mid-day, the temperature being 98.9° F. The autopsy disclosed pus on the right side between the cranial vault and the meninges up to the level of the convexity, towards the median region, and suppurative osteitis of the cranium. On opening the meninges a bed of very thick greenish-yellow pus (showing meningo-encephalitis) was laid bare. There was no lesion in the interior of the brain.—*New York Medical Journal.*

---

Unusual Effect of Cocaine.—Although, possibly, of no scientific importance, the following case is not without interest. It was presented to the Société de Chirurgie, and is reported in the *Medical Press:* M. Raymond presented a young girl, aged eighteen, who was found some time ago in a profound sleep in a railway wagon. The repeated efforts of the officials to awaken her proving unsuc-

---

[1] The assistant editor solicits contributions for this department,—new methods, new remedies and formulas, or any short practical note which may prove of value to the practitioner or student. Address 1338 Walnut Street, Philadelphia.

cessful, she was carried to the Salpêtrière Hospital. Here, it was only at the end of the third day that she was awakened artificially. The sleep was calm, the respiration and the pulse normal; the eyelids were closed, the face pale, and the muscular relaxation complete; in a word, the sleep was absolutely natural. When she came to herself she gave the following interesting account of her antecedents. At the beginning of the present year she went to a dentist to have a tooth pulled, and as she feared greatly the pain of the operation, the dentist employed cocaine. But after the tooth was drawn she was seized with convulsions, and finally fell into a deep sleep, and for three hours the dentist tried in vain to rouse her. Carried to the hospital, she awoke the following morning. For three months all was going on well, when one day she went to sleep in the train, and, carried again to the Lariboisière Hospital, she did not awaken until the seventh day. A few days after she was seized with sleep in the street, and was for the third time received at the hospital, where she remained asleep ten days. This time she remained under treatment three months. M. Raymond, after passing in review the possible causes of this phenomenon, said that he had fixed his opinion on hysteria, and gave the history of several other cases from a similar cause.—*Dental Record.*

